# Supplementary material for: Genome-wide screen for deficiencies modifying Cyclin G-induced developmental instability in Drosophila melanogaster
Source: Genetics. 2026 Jan 28;232(3):iyaf278. doi: 10.1093/genetics/iyaf278 (PMC13016881; doi:10.1093/genetics/iyaf278)
Supplement: iyaf278_Supplementary_Data [file iyaf278_supplementary_data.zip › Supplemental_Material_Legends_GENETICS-2025-308768.docx]

**Supplementary Figure 1:**

**a -** Position of the 15 landmarks on the wing. Landmarks 3 and 13 (red) were used to measure wing length.

**b –** Diagrams of primary and secondary screens.

*Df*: deficiency; *w^n^*: *w^5905^* or *w^6326^*; *Bal*: balancer chromosome; N: neutral deficiencies; D: deficiency that decreased *CycG^ΔP^-*induced fluctuating asymmetry; E: deficiency that increased *CycG^ΔP^-*induced fluctuating asymmetry.

**Supplementary Figure 2: Distributions of the FA fold-changes in the primary and secondary screens.**

**a –** FA fold-changes for the 452 deficiencies that produced measurable wings when combined with *da-Gal4*, *UAS-CycG^ΔP^*. Orange: FA fold-changes for the 39 deficiencies that significantly decreased *CycG^ΔP^*-induced FA. Green: FA of the 21 deficiencies that significantly increased *CycG^ΔP^*-induced FA. Grey: FA fold-changes for deficiencies that do not significantly modify *CycG^ΔP^*-induced FA. Black line: FA of the *da-Gal4*, *UAS-CycG^ΔP^* positive control.

**b –** FA fold-changes for the 68 deficiencies selected for the secondary screen. Green: three deficiencies showed a significantly higher FA compared to the genetic background. Grey: FA fold-changes for the remaining deficiencies. Black line: FA of the *w;+/da-Gal4* genetic background.

**c –** FA fold-changes of the 68 deficiencies selected for the secondary screen combined with *w; UAS-CycG^ΔP^/da-Gal4*. Orange: FA fold-changes of the 16 deficiencies that significantly decreased *CycG^ΔP^*-induced FA. Green: FA fold-changes for the 13 deficiencies that significantly increased *CycG^ΔP^*-induced FA. Grey: FA fold-changes for deficiencies that did not significantly modify *CycG^ΔP^*-induced FA. Black line: FA of the *da-Gal4*, *UAS-CycG^ΔP^* positive control.

**Supplementary Figure 3: Secondary screen – Mean wing size of flies heterozygous for the deficiencies associated with *da-Gal4* (top) or *da-Gal4,* *UAS-CycG^ΔP^* (down).**

Box-plots showing mean wing size. In grey, mean wing size of *+/da-Gal4* (**a**) or *+/da-Gal4, UAS-CycG^ΔP^* flies. In orange, mean wing size of deficiencies that decreased *CycG^ΔP^*-induced FA combined with *da-Gal4* (top) or *da-Gal4, UAS-CycG^ΔP^* (down). In green, mean wing size of deficiencies that increased *CycG^ΔP^*-induced FA combined with *da-Gal4* (top) *or da-Gal4, UAS-CycG^ΔP^* (**b**).

The two red lines correspond to the mean wing size of *+/da-Gal4* (1161) and *+/da-Gal4, UAS-CycG^ΔP^* (1014) flies.

*p<0.05, **p<0.01, ***p<0.001

**Supplementary Table 1: Deficiencies used in this study.**

**Supplementary Table 2: Alleles used in this study to identify genes that modify *CycG^ΔP^*-induced fluctuating asymmetry in the decreasing and enhancing deficiencies.**

**Supplementary Table 3: Secondary screen for deficiencies which modify *CycG^ΔP^*-induced FA.**

**Supplementary Table 4: Deficiencies used in Takahashi, Okada, Teramura, et al. (2011) that are shared or overlap with those used in the secondary screen.**
