# Supplementary material for: Genome-wide screen for deficiencies modifying Cyclin G-induced developmental instability in Drosophila melanogaster
Source: Genetics. 2026 Jan 28;232(3):iyaf278. doi: 10.1093/genetics/iyaf278 (PMC13016881; doi:10.1093/genetics/iyaf278)
Supplement: iyaf278_Supplementary_Data [file iyaf278_supplementary_data.zip › Supplementary_Figure_1_GENETICS-2025-308768.pdf]

a

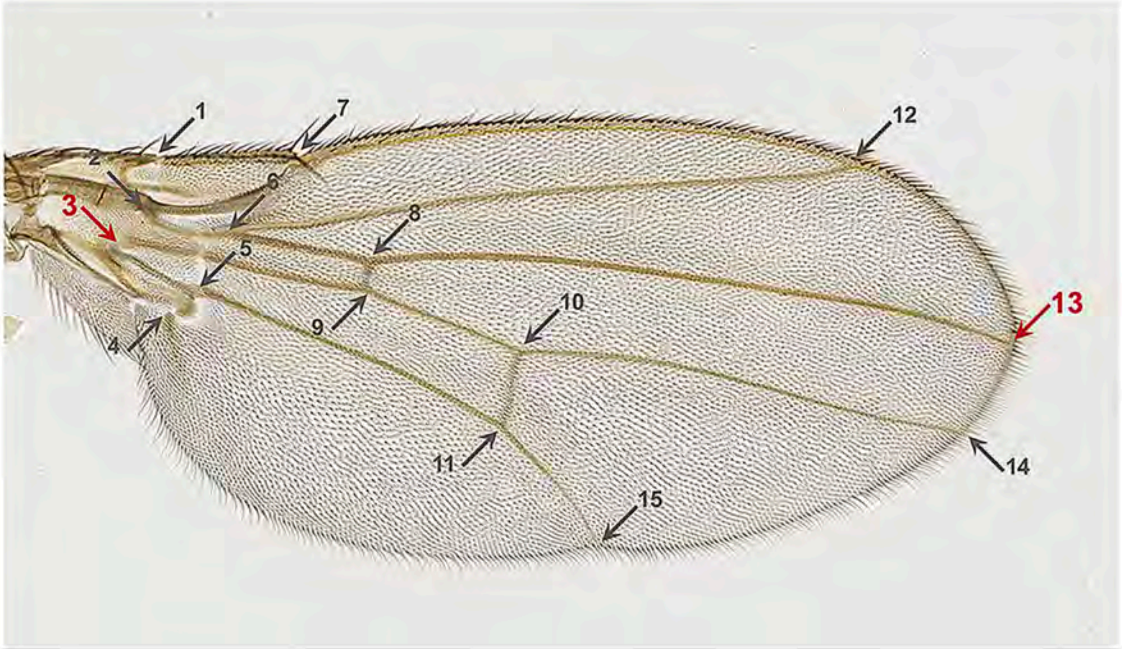

b

1 Primary screen

♀ w^n ; *Df/Bal* x ♂ *da-Gal4,UAS-CycG$^{\Delta P}$/TM6c,Sb* (452 *Df* tested – by series up to 25) ↓ 30 randomly selected ♀, wing length measurement ↓ FA (*Df-CycG$^{\Delta P}$*)	♀ w^n x ♂ *da-Gal4,UAS-CycG$^{\Delta P}$/TM6c,Sb* (one control per series) ↓ 30 randomly selected ♀, wing length measurement ↓ FA (*CycG$^{\Delta P}$*)

↙ No significant difference for 435 Df (N)

↘ Significant difference for 60 Df (E + D)

↓

Secondary screen

2 Secondary screen

♀ w^n ; *Df /Bal* x ♂ *da-Gal4,UAS-CycG$^{\Delta P}$/TM6c,Sb* (60 E or D Df + 8 N Df) ↓ 30 randomly selected ♀, wing length measurement ↓ FA (*Df-CycG$^{\Delta P}$*)	♀ w^n x ♂ *da-Gal4,UAS-CycG$^{\Delta P}$/TM6c,Sb* (one control per serie) ↓ 30 randomly selected ♀, wing length measurement ↓ FA (*CycG$^{\Delta P}$*)
♀ w^n ; *Df /Bal* x ♂ *da-Gal4* ↓ 30 randomly selected ♀, wing length measurement ↓ FA (Df)	♀ w^n x ♂ *da-Gal4* (one control per serie) ↓ 30 randomly selected ♀, wing length measurement ↓ FA (w^n)
