# Supplementary figures and images for: Genome-wide screen for deficiencies modifying Cyclin G-induced developmental instability in Drosophila melanogaster
Source: Genetics. 2026 Jan 28;232(3):iyaf278. doi: 10.1093/genetics/iyaf278 (PMC13016881; doi:10.1093/genetics/iyaf278)

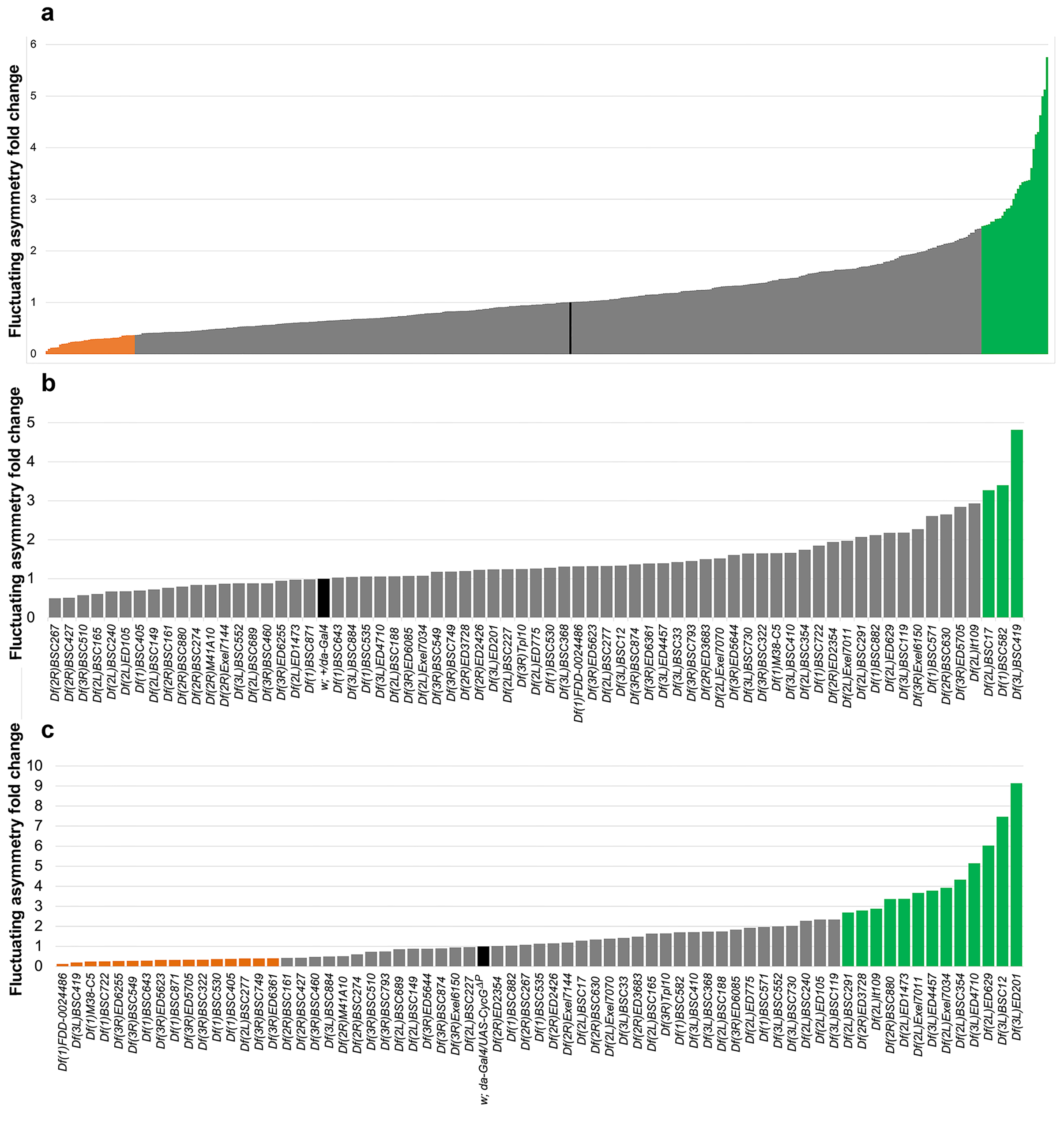

Supplement: iyaf278_Supplementary_Data [file iyaf278_supplementary_data.zip › Supplementary_Figure_2_GENETICS-2025-308768.tif]

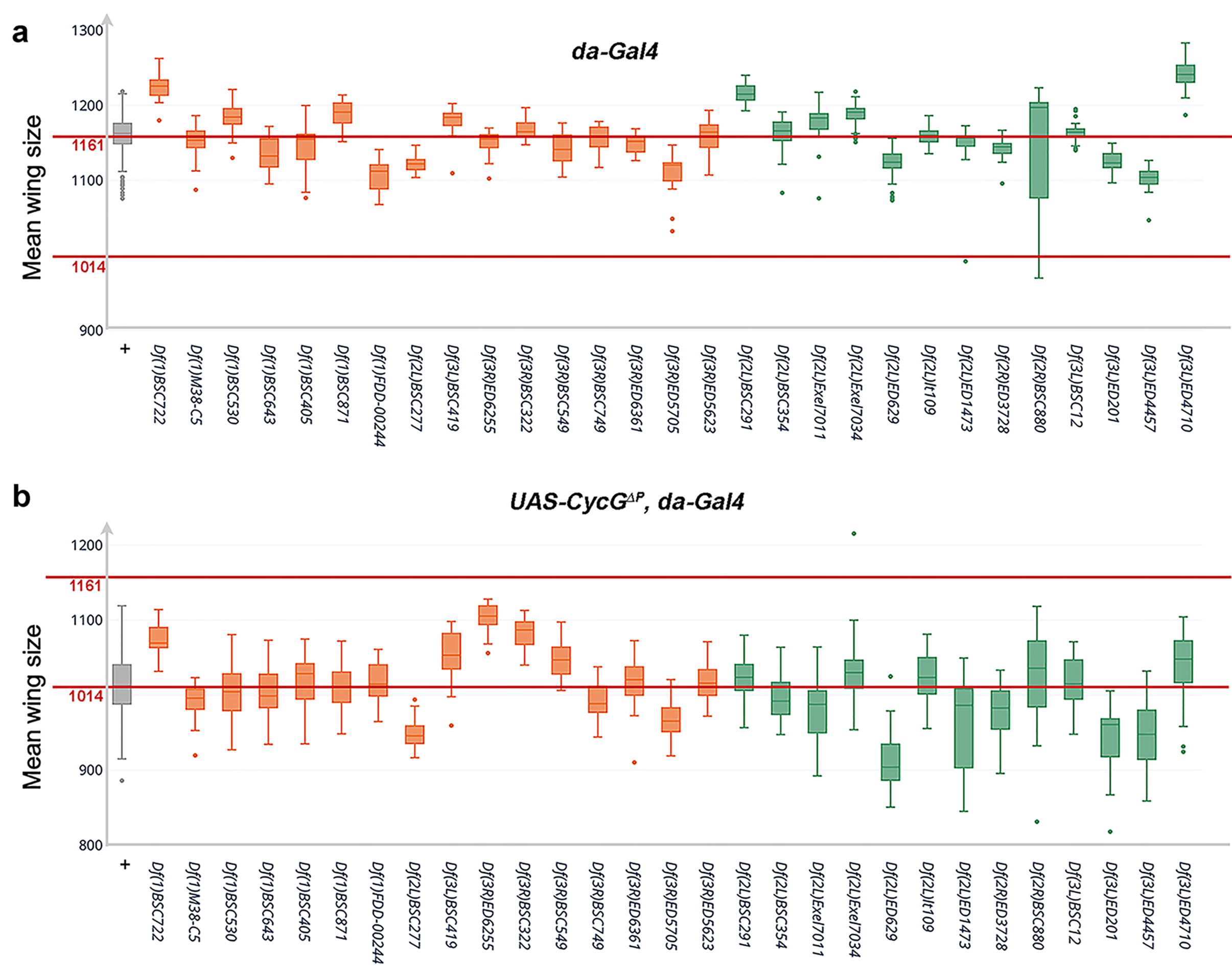

Supplement: iyaf278_Supplementary_Data [file iyaf278_supplementary_data.zip › Supplementary_Figure_3_GENETICS-2025-308768.tif]
